# TOP2A/MCM2, p16^INK4a^, and cyclin E1 expression in liquid-based cytology: a biomarkers panel for progression risk of cervical premalignant lesions

**DOI:** 10.1186/s12885-020-07740-1

**Published:** 2021-01-07

**Authors:** Oscar Del Moral-Hernández, Daniel Hernández-Sotelo, Luz del Carmen Alarcón-Romero, Miguel Angel Mendoza-Catalán, Eugenia Flores-Alfaro, Yaneth Castro-Coronel, Julio Ortiz-Ortiz, Marco Antonio Leyva-Vázquez, Carlos Ortuño-Pineda, Wendy Castro-Mora, Berenice Illades-Aguiar

**Affiliations:** 1 Laboratorio de Virología, Universidad Autónoma de Guerrero, Chilpancingo, Guerrero Mexico; 2 Laboratorio de Epigenética del Cáncer, Universidad Autónoma de Guerrero, Chilpancingo, Guerrero Mexico; 3 Laboratorio de Citopatología e Histoquímica, Universidad Autónoma de Guerrero, Chilpancingo, Guerrero Mexico; 4 Laboratorio de Biomedicina Molecular, Universidad Autónoma de Guerrero, Chilpancingo, Guerrero Mexico; 5 Laboratorio de Epidemiologia Clínica y Molecular de la Facultad de Ciencias Químico Biológicas, Universidad Autónoma de Guerrero, Chilpancingo, Guerrero Mexico; 6 Av. Lázaro Cárdenas S/N, Ciudad Universitaria, Facultad de Ciencias Químico Biológicas, 39090 Chilpancingo, Guerrero Mexico

**Keywords:** Cervical cancer, SIL, TOP2A/MCM2, p16^INK4a^, Cyclin E1, Biomarkers, HPV

## Abstract

**Background:**

To improve the efficiency of early diagnosis systems for cervical cancer, the use of cellular and viral markers for identifying precancerous lesions with a greater probability to progress to cancer has been proposed. Several cellular proteins and markers of oxidative DNA damage have been suggested as possible biomarkers of cervical carcinogenesis; however, they have not been evaluated together. In this study, we analyzed the expression of the cellular markers p16^INK4a^, Ki-67, CyclinE1, TOP2A/MCM2, and telomerase, as well as the DNA oxidative damage markers ROS and 8-OHdG. The analyses were performed in liquid-based cervical cytology samples or biopsies with premalignant lesions or cervical cancer diagnosis, with the purpose of selecting a panel of biomarkers that allow the identification of precursor lesions with greater risk of progression to cervical cancer.

**Methods:**

We analyzed 1485 liquid-based cytology samples, including 239 non-squamous intraepithelial lesions (NSIL), 901 low-grade squamous intraepithelial lesions (LSIL), 54 high-grade squamous intraepithelial lesions (HSIL), and 291 cervical cancers (CC). The biomarkers were analyzed by immunocytochemistry and Human Papilloma Virus (HPV) genotyping with the INNO-LiPA genotyping Extra kit.

**Results:**

We found that all tested cellular biomarkers were overexpressed in samples with high risk-HPV infection, and the expression levels increased with the severity of the lesion. TOP2A/MCM2 was the best biomarker for discriminating between LSIL and HSIL, followed by p16^INK4a^ and cyclinE1. Statistical analysis showed that TOP2A/MCM2 provided the largest explanation of HSIL and CC cases (93.8%), followed by p16^INK4a^ (91%), cyclin E1 (91%), Ki-67 (89.3%), and telomerase (88.9%).

**Conclusions:**

We propose that the detection of TOP2A/MCM2, p16^INK4a^ and cyclin E1 expression levels is useful as a panel of biomarkers that allow identification of cervical lesions with a higher risk for progression to CC with high sensitivity and precision; this can be done inexpensively, in a single and non-invasive liquid-based cytology sample.

**Supplementary Information:**

The online version contains supplementary material available at 10.1186/s12885-020-07740-1.

## Background

Cervical cancer (CC) is the fourth leading cause of cancer-related death in women worldwide, with an estimated 528,000 new cases and 266,000 deaths in 2012. In Mexico, CC is the second most common type of cancer in women, and it shows a variable distribution. In 2013, 13,960 new cases and 4769 deaths were reported in Mexico. In southern Mexico, the CC mortality rate is 14.2 per 100,000 women affected, which is higher than the national average [1].

The primary cause of CC is persistent infection with high-risk human papillomavirus (HR-HPV) [2]. The reasons that most patients remain asymptomatic and eliminate HPV infections whereas other asymptomatic infections progress to precancerous lesions are poorly understood. The possible reasons include factors inherent to the host, such as immune response, genetic risk factors, and lifestyle, and virus-related factors, such as differences in virus genomes and viral load [3, 4].

The Pap smear and colposcopy are the most common options for timely CC diagnosis around the world. However, large numbers of false negatives and false positives have led to over-intervention, with negative consequences treated women [5]. The introduction of HPV DNA detection tests has successfully improved the prospects for prevention. However, one disadvantage of these tests is that they do not distinguish between asymptomatic transient infections and persistent carcinogenic infections [6]. To improve the efficiency of early diagnosis programs for CC, the use of cellular and viral markers has been proposed to increase the sensitivity of screening and reduce the false-negative rate. Several biomarkers have been suggested, including p16^INK4A^, [7], Ki-67 [8], proliferating cell nuclear antigen (PCNA) [9], p21, cyclin-D, cyclin-E [8], minichromosome maintenance protein-2 (MCM2) and DNA Topoisomerase II α (TOP2A) [10, 11], and telomerase [12].

The p16^INK4a^ protein is a tumor suppressor that inhibits CDK4 and CDK6. In differentiated epithelial cells, p16^INK4a^ expression is not detected; however, in dysplastic cervical epithelial cells and HPV-positive CC cells, p16^INK4a^ is overexpressed [7]. Another marker of cell proliferation is Ki-67, which is only expressed in growing cells [13]. In addition, overexpression of MCM2 and TOP2A has been reported as a potential diagnostic biomarker in CC [11]. MCM2 is overexpressed in CC, whereas in the normal cervical epithelium, it is only detected in the basal proliferating layer [14]. TOP2A is a nuclear protein that controls DNA topology during DNA replication and chromosome separation, and its overexpression is associated with the progression from cervical intraepithelial neoplasia grade 2 to more advanced cervical lesions [15]. Amplification of human telomerase is known to be associated with cervical tumorigenesis [16], although its role in the progression of cervical lesions is still unclear.

There are other cellular biomarkers, such as reactive oxygen species (ROS). A well-known marker of ROS-induced oxidative DNA damage is 8-hydroxydeoxyguanosine (8-OHdG). It has been reported that there is a link between oxidative DNA damage and the progression of cervical dysplasia [17]. Cellular biomarkers are needed to improve the diagnostic sensitivity of cervical premalignant lesions along with HPV-type detection in a single, economic, liquid-based cytology sample. In this study, we analyzed a set of cellular biomarkers in premalignant cervical lesions and CC and selected a panel that efficiently identifies lesions that are likely to progress to CC.

## Methods

### Sample collection

All analyzed samples were cervical scrapings or biopsies obtained from women in southern Mexico collected in 2013–2016. All study participants provided written informed consent and responded to a questionnaire with socio-demographic, clinical, and obstetrical information. The cervical scrapes were obtained from women who utilized the Cervical Cancer Screening Service of the Facultad de Ciencias Químico Biológicas of the Universidad Autónoma de Guerrero, and the biopsies were obtained from of the Hospital General “Dr. Raymundo Abarca Alarcón” in Chilpancingo, and from Instituto Estatal de Cancerología “Dr. Arturo Beltrán Ortega” in Acapulco, Guerrero, Mexico. The Bioethical Committee of the Universidad Autónoma de Guerrero approved this study.

### Cytological and histopathological diagnosis

A total of 1485 cervical cytology samples from women aged 26–66 were analyzed, which included 239 samples without intraepithelial squamous lesion (NSIL), 901 low-grade intraepithelial squamous lesions (LSIL), 54 high-grade intraepithelial squamous lesions (HSIL), and 291 CCs. Cervical specimens were obtained by liquid-based cytology (liquid-PREP™) and smears were subjected to cytomorphological examination using Papanicolaou [18] and were read by an experienced cytopathologist and classified according to the Bethesda system. Sampling for the cytological study was directed by a colposcope. A scrape was taken from the squamocolumnar transformation zone for later analysis, and from the same anatomical site, a biopsy was taken to confirm the diagnosis by histopathology (HSIL and CC). Histological diagnosis was defined according to the classification system of the International Federation of Gynecology and Obstetrics [19].

### HPV detection and typing

DNA was extracted using the standard SDS-proteinase K-phenol-chloroform method [20]. HPV was detected and typed with INNO-LiPA Genotyping Extra software (Innogenetics), which allows the identification of 28 HPV low- and high-risk genotypes [21].

### Cellular biomarker detection

The expression of the biomarkers p16^INK4^, Ki-67, cyclin-E, TOP2A/MCM2, telomerase, and 8-OHdG was determined by the streptavidin-biotin-peroxidase immunocytochemical method, using the Cytoscan HRP detection system (Cell Marque Corporation, Hot Springs, AR, USA). Cytology slides in a liquid base were subjected to antigen retrieval (immuno DNA retrieval with citrate; Bio SB Inc., Santa Barbara, CA, USA) for 5 min at 120 °C. The monoclonal antibodies used were anti-p16^INK4a^ (E6H4; CINtec; ROCHE, Switzerland), Ki-67 (Dako, Carpinteria, CA, USA), telomerase (2C4; Novus Biologicals, Littleton, CO, USA), cyclin-E (13A3; Novocastra, Newcastle-Upon-Tyne, UK), and topoisomerase II α/MCM2 (Santa Cruz Biotechnology, Santa Cruz, CA, USA). The slides were incubated with the primary antibodies for 1 h, with the biotin-conjugated secondary antibody for 20 min, and then with streptavidin peroxidase. The reaction was developed with chromogen diaminobenzidine DAB, and samples were counterstained with Mayer’s hematoxylin. The cells were imaged at 40X magnification on the Leica application suite v3.3.0 using a LEICA-DM1000 microscope equipped whit a EC3 camera (Leica microsystems, Switzerland). Protein expression was scored as follows: negative, 0%; mild, 1–11%; moderate, 12–50%; and intense, > 50% [22, 23]. HeLa cells were used as positive controls. To evaluate ROS levels, the CellROX Oxidative Stress Reagents kit (Thermo Scientific, USA) was used according to the manufacturer’s instructions, and was subsequently analyzed using flow cytometry in a FACSCanto II (BD) instrument.

### Statistical analysis

We summarized the socio-demographic information and risk factors as means for quantitative variables and as frequencies for qualitative variables. One-factor analysis of variance and the chi-square test (X^2^ test) were used to compare means, and Fisher’s exact test was used to compare frequencies. To construct risk indices and determine the correlations between the expression levels of different cell markers, principal component analysis (PCA) was performed, and from this analysis, the reliability coefficient Cronbach’s alpha was obtained. The factor extracted from the PCA was compared to the average standardized expression levels (Z score) of the markers. Therefore, the expression levels of the markers were standardized to construct risk indices for five, four, three, or two markers. To estimate the effect of a single marker and risk index on the probability of LSIL, HSIL or CC, multinomial logistic regression models adjusted for age and HPV stratified by oncogenic risk were used. Odds ratios and confidence intervals at 95% were calculated. The statistical analysis was performed using STATA 13.0 (Stata Corporation, College Station, TX, USA).

## Results

A total of 1485 samples was included, which included 239 NSIL, 901 LSIL, 54 HSIL, and 291 CC samples. The mean age of the study subjects was 39.6 ± 11.3 years (range 19–74) for those with NSIL samples, 37.4 ± 11.6 years (range 14–82) for those with LSIL, 38.4 ± 12.4 years (range 20–63) for those with HSIL, and 53.1 ± 13.2 years (range 24–89) for those with CC. The main socio-demographic and sexual conduct characteristics associated with SIL and CC are shown in Table 1. The age, alcohol consumption, parity, sexual age at screening, number of lifetime sexual partners, and years of education were found to be statistically significant factors for NSIL, LSIL, HSIL, and CC.

**Table 1:** Socio-demographics and sexual conduct of study subjects according to cervical screening results

	NSIL		LSIL		HSIL		CC		
	*n* = 239	%	*n* = 901	%	*n* = 54	%	*n* = 291	%	*p*
Age (years)^a^	39.8 ± 11.3		37.4 ± 11.6		38.4 ± 12.5		53.1 ± 13.2		0.001^b^
Range	19–74		14–82		20–63		24–89		
Smoking status									
No	204	85.4	785	87.1	46	85.2	189	64.9	0.905^c^
Yes	23	9.6	88	9.8	6	11.1	25	8.6	
Unknown	12	5.0	28	3.1	2	3.7	77	26.5	
Alcohol consumption									
No	119	49.8	563	62.5	41	75.9	173	59.4	0.001^c^
Yes	102	42.7	296	32.7	11	20.4	20	6.9	
Unknown	18	7.5	43	4.8	2	3.7	98	33.7	
Parity									
None	41	17.1	196	21.7	8	14.8	4	1.4	0.001^c^
1–2	82	34.3	354	39.3	10	18.6	18	6.2	
3–5	97	40.6	288	32.0	14	25.9	83	28.5	
≥ 6	15	6.3	53	5.9	18	33.3	134	46.0	
Unknown	4	1.7	10	1.1	4	7.4	52	17.9	
Sexual age at screening									
<16	12	5.1	75	8.3	11	20.4	87	29.9	0.001^c^
16–20	131	54.8	486	53.9	33	61.1	115	39.5	
>20	94	39.3	335	37.2	8	14.8	32	11.0	
Unknown	2	0.8	5	0.6	2	3.7	57	19.6	
No. of life time sexual partners									
1-2	183	76.6	644	71.5	42	77.8	170	58.4	0.015^c^
≥3	44	18.4	224	24.9	8	14.8	36	12.4	
Unknown	12	5.0	33	3.6	4	7.4	85	29.2	
Education (years)									
0	0	0.0	20	2.2	9	16.7	79	27.1	0.001^c^
6	24	10.0	125	13.9	23	42.6	78	26.8	
9	24	10.0	131	14.5	2	3.7	8	2.8	
10–12	151	63.2	510	56.6	16	29.6	3	1.0	
≥13	23	9.6	79	8.8	0	0.0	0	0.0	
Unknown	17	7.2	36	4.0	4	7.4	123	42.3	

*NSIL* non-squamous intraepithelial lesions, *LSIL* low-grade squamous intraepithelial lesions, *HSIL* high-grade squamous intraepithelial lesions, *CC* cervical cancer. ^a^Expressed as mean ± standard deviation; ^b^Kruskal-Wallis; ^c^Fisher’s exact test

### HPV-16, − 18 and − 45 are the genotypes most frequent in cervical cancer cases

The prevalence of HPV infection was 69.1% in NSIL, 99.9% in LSIL, 100% in HSIL, and 98.3% in CC. Single HPV infection was most common among all samples, with prevalence rates of 50.8% in NSIL, 61.2% in LSIL, 44.6% in HSIL, and 84.3% in CC (Table 2). Among samples with single infections, HR-HPVs were the most prevalent (NSIL, 25.5%; LSIL, 25.6%; HSIL, 31.5%; and CC, 65.3%). Multiple HPV infections were detected in 3.3% of NSIL samples, 9.9% of LSIL samples, 22.1% of HSIL samples, and 7.7% of CC samples. Mixed HPV infections were detected in 15% of NSIL samples, 28.8% of LSIL samples, 33.3% of HSIL samples, and 6.3% of CC samples (Table 2). We found that the most frequent HR-HPV genotypes in CC cases were 16 (42.3%), 18 (7.9%), and 45 (4.5%), followed by 52 and 69 (1.4%) (Table 3).

**Table 2:** Prevalence of single, multiple, and mixed-HPV infections in cervix without SIL, with SIL and CC

	NSIL		LSIL		HSIL		CC	
	*n* = 239	%	*n* = 901	%	*n* = 54	%	*n* = 291	%
HPV negative	74	30.9	1	0.1	0	0.0	5	1.7
HPV positive	165	69.1	900	99.9	54	100	286	98.3
**Single HPV infection**								
HR	61	25.5	231	25.6	17	31.5	190	65.3
PHR	16	6.8	78	8.7	3	5.7	2	0.7
LR	19	7.9	95	10.5	0	0.0	2	0.7
UR	25	10.6	148	16.4	4	7.4	51	17.6
Total	121	50.8	552	61.2	24	44.6	245	84.3
**Multiple HPV infection**								
HR	5	2.1	65	7.2	10	18.5	20	7.0
PHR	1	0.4	11	1.2	0	0.0	0	0.0
LR	1	0.4	7	0.8	1	1.8	2	0.7
UR	1	0.4	6	0.7	1	1.8	0	0.0
Total	8	3.3	89	9.9	12	22.1	22	7.7
**Mixed HPV infection**								
HR and PHR	11	4.6	66	7.4	4	7.5	1	0.3
HR and LR	11	4.6	86	9.5	6	11.1	13	4.5
HR and UR	2	0.8	18	2.0	4	7.5	1	0.3
PHR and LR	4	1.7	26	2.9	1	1.8	1	0.3
PHR and UR	2	0.8	10	1.2	0	0.0	1	0.3
LR and UR	0	0.0	8	0.9	0	0.0	1	0.3
HR, PHR, and LR	4	1.7	31	3.4	0	0.0	1	0.3
HR, PHR, and UR	0	0.0	4	0.4	1	1.8	0	0.0
HR, LR, and UR	1	0.4	7	0.8	1	1.8	0	0.0
PHR, LR, and UR	1	0.4	0	0.0	0	0.0	0	0.0
HR, PHR, LR, and UR	0	0.0	3	0.3	1	1.8	0	0.0
Total	36	15.0	259	28.8	18	33.3	19	6.3

*NSIL* Non-squamous intraepithelial lesions, *LSIL* Low-grade squamous intraepithelial lesions, *HSIL* High-grade squamous intraepithelial lesions, *CC* Cervical cancer, *HR* High risk, *PHR* Probably high risk, *LR* Low risk, *UR* Undeterminate risk, *Single HPV* Infection with one genotype, *Multiple HPV* Infection with two genotypes of the same-risk group, *Mixed HPV* Infection with genotypes of different oncogenic risk groups

**Table 3:** HPV genotypes in single, multiple, and mixed-HPV infections in cervix without SIL, with SIL and CC

	O-Risk	NSIL		LSIL		HSIL		CC	
		*n* = 239	%	*n* = 901	%	*n* = 54	%	*n* = 291	%
HPV negative		74	30.9	1	0.1	0	0.0	5	1.7
**Single HPV infection**									
16	HR	40	16.7	119	13.2	7	13.0	123	42.3
18	HR	4	1.7	13	1.4	3	5.6	23	7.9
51	HR	6	2.5	19	2.1	1	1.8	0	0.0
52	HR	2	0.8	19	2.1	0	0.0	4	1.4
45	HR	1	0.4	7	0.8	1	1.8	13	4.5
66	PHR	7	2.9	41	4.4	2	3.7	0	0.0
53	PHR	9	3.8	23	2.5	1	1.8	0	0.0
68	PHR	0	0.0	10	1.1	0	0.0	1	0.3
26	PHR	0	0.0	2	0.2	0	0.0	1	0.3
83	PHR	0	0.0	3	0.3	0	0.0	0	0.0
6	LR	15	6.3	62	6.9	0	0.0	1	0.3
70	LR	1	0.4	8	0.9	0	0.0	0	0.0
11	LR	0	0.0	7	0.8	0	0.0	1	0.3
54	LR	0	0.0	8	0.9	0	0.0	0	0.0
44	LR	2	0.8	5	0.5	0	0.0	0	0.0
74	UR	2	0.8	10	1.1	0	0.0	0	0.0
62	UR	0	0.0	4	0.4	0	0.0	0	0.0
69	UR	0	0.0	0	0.0	0	0.0	4	1.4
89	UR	0	0.0	2	0.2	0	0.0	0	0.0
67	UR	0	0.0	0	0.0	0	0.0	1	0.3
**Multiple HPV infection**									
16 and 52	HR	0	0.0	7	0.8	1	1.8	6	2.0
31 and 33	HR	1	0.4	6	0.7	4	7.4	3	1.0
16 and 18	HR	0	0.0	8	0.9	1	1.8	4	1.4
16 and 56	HR	1	0.4	4	0.4	1	1.8	0	0.0
16 and 39	HR	0	0.0	2	0.2	0	0.0	2	0.7
53 and 66	PHR	1	0.4	6	0.7	0	0.0	0	0.0
58 and 68	PHR	0	0.0	2	0.2	0	0.0	0	0.0
66 and 53	PHR	0	0.0	2	0.2	0	0.0	0	0.0
66 and 68	PHR	0	0.0	1	0.1	0	0.0	0	0.0
11 and 54	LR	1	0.4	1	0.1	0	0.0	0	0.0
6 and 11	LR	0	0.0	0	0.0	1	1.8	0	0.0
6 and 54	LR	0	0.0	1	0.1	0	0.0	0	0.0
6 and 7	LR	0	0.0	1	0.1	0	0.0	0	0.0
6 and 70	LR	0	0.0	1	0.1	0	0.0	0	0.0
69 and 71	UR	1	0.4	3	0.3	1	1.8	0	0.0
**Mixed HPV infection**									
16 and 6	HR, LR	4	1.7	20	2.2	1	1.8	3	1.0
53 and 6	PHR, LR	0	0.0	8	0.9	0	0.0	0	0.0
16 and 53	HR, PHR	1	0.4	6	0.7	0	0.0	0	0.0
66 and 6	PHR, LR	0	0.0	6	0.7	0	0.0	1	0.3
39 and 6	HR, LR	0	0.0	4	0.4	0	0.0	2	0.7
68 and 6	PHR, LR	1	0.4	5	0.5	0	0.0	0	0.0
16 and 68	HR, PHR	0	0.0	5	0.5	0	0.0	0	0.0
16 and 66	HR, PHR	0	0.0	4	0.4	0	0.0	0	0.0
18 and 6	HR, LR	0	0.0	3	0.3	0	0.0	1	0.3

*O-Risk* Oncogenic risk, *NSIL* Non-squamous intraepithelial lesions, *LSIL* Low-grade squamous intraepithelial lesions, *HSIL* High-grade squamous intraepithelial lesions, *CC* Cervical cancer, *HR* High risk, *PHR* Probably high risk, *LR* Low risk, *UR* Indeterminate risk. *Single HPV* Infection with one genotype (shown are the five most prevalent genotypes by risk group), *Multiple HPV* Infection with two genotypes of the same risk group (shown are the most prevalent genotypes by risk group), *Mixed HPV* Infection with genotypes of different risk groups (shown are the most prevalent combinations)

### TOP2A/MCM2, p16^INK4a^ and cyclin-E expression is associated with the progression to CC

The expression of cellular markers was significantly higher in CC than in HSIL, LSIL, and NSIL (Table 4), which suggest that expressions of all tested cellular markers increase according to cervical lesion severity. On the other hand, the levels of 8-OHdG and ROS were significantly higher in LSIL than NSIL; however, these levels apparently did not increase together with cervical lesion severity, and the ROS level decreased as the cervical lesion progressed (Table 4, Additional file 1: Table S1). The PCA identified a single component with a percent explanation of 82.7, and a Kaiser–Meyer–Olkin test value of 0.905; this component grouped TOP2A/MCM2, p16^INK4a^, cyclin-E, Ki-67, and telomerase, and we found in the analysis that the level of TOP2A/MCM2 expression provided the largest explanation (93.8%) of the five included markers, followed by p16^INK4a^ and cyclin-E (both 91%), Ki-67 (89.3%), and telomerase (88.9%) (Additional file 2: Table S2), which indicates that an increase in the expression of these five cellular markers (mainly TOP2A/MCM2) was statistically related to the development and progression of cervical lesions in the studied population. Notably, the expression of the cellular markers was highly correlated, with a Cronbach’s alpha reliability coefficient of 0.949. By contrast, when 8-OHdG and ROS were added to the statistical model, a poor or non-existent correlation with the other cellular markers was observed. These observations suggest that expressions of the cellular markers TOP2A/MCM2, p16^INK4a^, cyclin-E, Ki-67, and telomerase are biologically related, whereas ROS and 8-OHdG expressions behave differently and appear independent from the expression of the cellular markers.

**Table 4:** Expression of cellular markers in normal cervix, SIL, and cervical cancer

	NSIL		LSIL		HSIL		CC		
	*n* = 79	%	*n* = 208	%	*n* = 35	%	*n* = 42	%	*p*
**p16INK4a**									
Negative	33	41.8	0	0.0	0	0.0	0	0.0	< 0.001
Mild	29	36.7	7	3.4	1	2.9	0	0.0	
Moderate	11	13.9	168	80.8	6	17.1	0	0.0	
Intense	6	7.6	33	15.9	28	80.0	42	100	
**Ki-67**									
Negative	33	41.8	2	1.0	0	0.0	0	0.0	< 0.001
Mild	28	35.4	26	12.5	2	5.7	0	0.0	
Moderate	18	22.8	165	79.3	8	22.9	0	0.0	
Intense	0	0.0	15	7.2	25	71.4	42	100	
**Cyclin E**									
Negative	36	45.6	1	0.5	0	0.0	0	0.0	< 0.001
Mild	27	34.2	17	8.2	0	0.0	0	0.0	
Moderate	13	16.4	170	81.7	14	40.0	0	0.0	
Intense	3	3.8	20	9.6	21	60.0	42	100	
**TOP2A/MCM2**									
Negative	42	53.2	0	0.0	0	0.0	0	0.0	< 0.001
Mild	25	31.6	20	9.6	0	0.0	0	0.0	
Moderate	11	13.9	179	86.1	11	31.4	0	0.0	
Intense	1	1.3	9	4.3	24	68.6	42	100	
**Telomerase**									
Negative	28	35.4	1	0.5	0	0.0	0	0.0	< 0.001
Mild	31	39.2	8	3.8	0	0.0	0	0.0	
Moderate	19	24.1	124	59.6	8	22.9	0	0.0	
Intense	1	1.3	75	36.1	27	77.1	42	100	
**ROS**									
Q1	38	29.5	38	17.9	5	17.2	17	53.1	< 0.001
Q2	45	34.9	37	17.5	9	31.1	10	31.3	
Q3	30	23.2	55	25.9	12	41.4	4	12.5	
Q4	16	12.4	82	38.7	3	10.3	1	3.1	
**8-OHdG**									
Q1	63	67.7	11	6.5	5	19.2	0	0	< 0.001
Q2	23	24.7	40	23.5	6	23.1	12	37.5	
Q3	4	4.3	67	39.4	5	19.2	5	15.6	
Q4	3	3.2	52	30.6	10	38.5	15	46.9	

*NSIL* Non-squamous intraepithelial lesions, *LSIL* Low-grade squamous intraepithelial lesions, *HSIL* High-grade squamous intraepithelial lesions, *CC* Cervical cancer; 8-OHdG and ROS levels are expressed as quartiles, Q1-Q4. Interquartile range

*P* values were calculate using X^2^ test

**Fig. 1:**
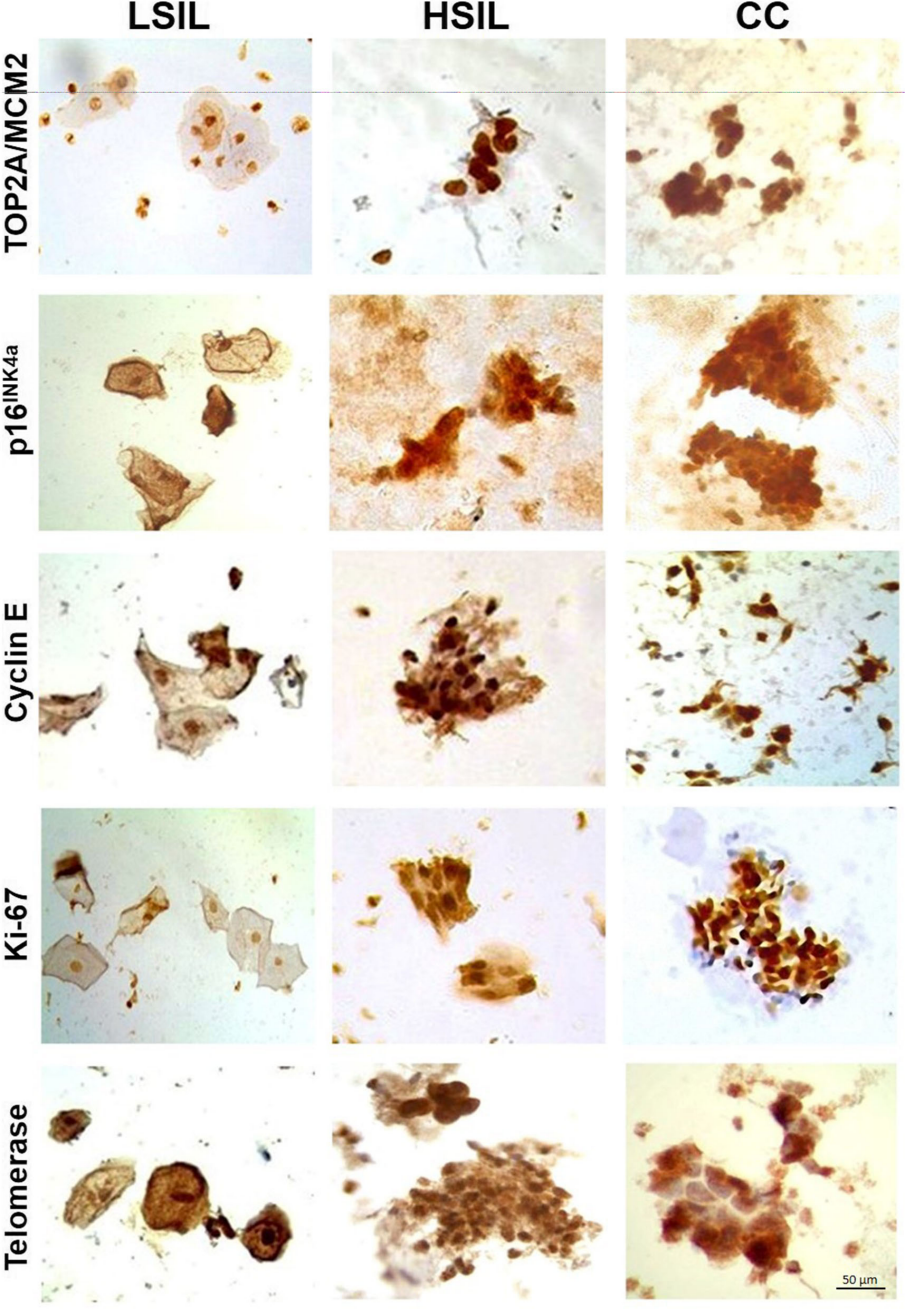
Expression of cellular biomarkers p16^INK4^, Ki-67, Cyclin-E, TOP2A/MCM2, and telomerase in LSIL and cervical cancer. Representative images of liquid-based cytology sample. LSIL, low-grade squamous intraepithelial lesions; HSIL, high-grade squamous intraepithelial lesions; CC, cervical cancer. 40X magnification; Scale bar represents 50 μm

The expression of TOP2A/MCM2, p16^INK4a^, cyclin-E, Ki-67, and telomerase increased in HSIL and CC compared to LSIL cases, which was evident through immunocytochemistry in cervical scrapings (Fig. 1). Moreover, in LSIL samples, the subcellular location was both nuclear and cytoplasmatic for p16^INK4a^, cyclin-E, and telomerase, while TOP2A/MCM2 and Ki67 were observed exclusively in nuclei. By contrast, in HSIL and CC cases the cell markers were in both nuclei and cytoplasm, except for TOP2A/MCM2, which remained exclusively nuclear, but with a much greater intensity than LSIL (Fig. 1).

Using adjusted multinomial logistic regression models Individually, we evaluated the association of the cellular markers’ expression with LSIL, HSIL, and CC diagnoses. Singly, the increase in the expression of the five abovementioned cellular markers was associated with LSIL, HSIL, and CC development (Table 5). However, the increased expression of the five cellular markers analyzed together (RI-5, obtained through the PCA analysis), was strongly associated with the risk of LSIL, HSIL, and CC development. The results indicate that an increase in TOP2A/MCM2, p16^INK4a^, cyclin-E, Ki-67, and telomerase expression confers a greater joint risk to develop CC (OR = 8290, CI: 1309-∞), HSIL (OR = 4012, CI: 755–21,323), and LSIL (OR = 58, CI: 18.5–186.1) compared to the NSIL group (Table 5, Additional file 3: Table S3). A similar effect was observed when the progression from LSIL to HSIL and CC and from HSIL to CC was analyzed; however, the association increased by only grouping TOP2A/MCM2, p16^INK4a^, and cyclin-E (RI-3, obtained by the PCA analysis). Increased expression of TOP2A/MCM2, p16^INK4a^, and cyclin-E led to 79.1- and 246.1-fold increases in the progression risks to HSIL and CC, respectively, and a 2.8-fold increase progression risk of HSIL to CC (Table 6). Overall, our results suggest that the cellular markers TOP2A/MCM2, p16^INK4a^, and cyclin-E could be associated with the development and progression of cervical lesions, while ROS and 8-OHdG could be related to the development of lesions but may not be determinant in the progression of cervical lesions. Therefore, TOP2A/MCM2, p16^INK4a^, and cyclin-E expression, determined in a single cervical sample, could be useful for determining the prognosis of premalignant cervical lesions.

**Table 5:** Cellular biomarkers and oxidative damage and their association to SIL and cervical cancer development

	NSIL^**a**^	LSIL			HSIL			CC		
	OR	OR	CI	***p***	OR	CI	***p***	OR	CI	***p***
**TOP2A/MCM2**	1.0	22.5	9.3–54.1	<0.001	900.8	240.3–3376	<0.001	2484	507–∞	<0.001
**p16INK4a**	1.0	10.1	4.6–22.2	<0.001	138.1	42.6–447.8	<0.001	320.6	75.0–∞	<0.001
**Cyclin E**	1.0	10.0	4.6–21.3	<0.001	109.9	36.3–333.2	<0.001	533.0	123.3–∞	<0.001
**Ki-67**	1.0	6.7	3.4–13.2	<0.001	121.4	40.9–360.3	<0.001	522.8	121.5–∞	<0.001
**Telomerase**	1.0	13.7	6.1–30.7	<0.001	85.7	26.2–280.5	<0.001	165.9	39.2–∞	<0.001
**RI-5**	1.0	58	18.5–186.1	<0.001	4012	755–21,323	<0.001	8290	1309–∞	<0.001
**RI-4**	1.0	29.5	11.3–76.8	<0.001	1482	340.8–6461	<0.001	3878	691–∞	<0.001
**RI-3**	1.0	40.2	14.2–114.4	<0.001	2924	592–14,429	<0.001	5913	998–∞	<0.001
**RI-2**	1.0	24.5	9.9–60.8	<0.001	1290	306.8–5429	<0.001	3020	526–∞	<0.001
**ROS**	1.0	1.8	1.4–2.3	<0.001	1.2	0.8–1.8	0.30	0.5	0.3–0.8	0.004
**8-OHdG**	1.0	4.4	2.7–7.2	<0.001	3.6	1.9–6.6	<0.001	5.3	2.8–10.0	<0.001

*NSIL* Non-squamous intraepithelial lesions, *LSIL* Low-grade squamous intraepithelial lesions, *HSIL* High-grade squamous intraepithelial lesions, *CC* Cervical cancer, *OR* Odds ratio, *CI* Confidence interval, *RI* Risk index; RI-5 analysis with TOP2A/MCM2, p16INK4a, cyclin E, Ki-67, and telomerase; RI-4 analysis with TOP2A/MCM2, p16INK4a, cyclin E, and Ki-67; RI-3 analysis with TOP2A/MCM2, p16INK4a, and cyclin E; RI-2 analysis with TOP2A/MCM2 and p16INK4a OR adjusted by age and HPV infection by oncogenic risk (HPV negative, HR, PHR, LHR, and UHR) ^a^reference category

**Table 6:** Cellular biomarkers and oxidative damage and their association to HSIL and cervical cancer development

	LSIL^**a**^	HSIL			CC			HSIL^**a**^	CC		
	OR	OR	CI	***p***	OR	CI	***p***	OR	OR	CI	***p***
**TOP2A/MCM2**	1.0	44.9	16.6–121.4	< 0.001	167.4	43.1–∞	< 0.001	1.0	2.6	0.6–∞	0.183
**p16INK4a**	1.0	16.2	6.7–38.9	< 0.001	51.3	14.4–∞	< 0.001	1.0	1.9	0.5–∞	0.312
**Cyclin E**	1.0	13.1	5.8–29.9	< 0.001	78.5	21.7–∞	< 0.001	1.0	3.9	0.9–∞	0.060
**Ki-67**	1.0	17.7	7.5–41.4	< 0.001	89.0	24.1–∞	< 0.001	1.0	2.4	0.7–∞	0.146
**Telomerase**	1.0	6.1	2.5–14.6	< 0.001	15.6	4.5–∞	< 0.001	1.0	1.4	0.4–∞	0.562
**RI-5**	1.0	72.7	21.8–242.2	< 0.001	261	56.5–∞	< 0.001	1.0	2.8	0.7–∞	0.152
**RI-4**	1.0	55.2	18.1–168.3	< 0.001	203.3	47.3–∞	< 0.001	1.0	3.0	0.8–∞	0.110
**RI-3**	1.0	79.1	23.7–264.4	< 0.001	246.1	55.8–∞	< 0.001	1.0	2.8	0.7–∞	0.140
**RI-2**	1.0	61.4	20.0–188.8	< 0.001	200.8	44.1–∞	< 0.001	1.0	2.2	0.6–∞	0.224
**ROS**	1.0	0.7	0.5–1.0	0.055	0.3	0.2–0.5	0.30	1.0	0.4	0.2–0.7	0.003
**8-OHdG**	1.0	0.8	0.5–1.2	0.321	1.2	0.7–1.9	0.487	1.0	1.2	0.7–2.1	0.415

*LSIL* Low-grade squamous intraepithelial lesions, *HSIL* High-grade squamous intraepithelial lesions, *CC* Cervical cancer, *OR* Odds ratio, *CI* Confidence interval, *RI* Risk index; RI-5 analysis with TOP2A/MCM2, p16INK4a, cyclin E, Ki-67, and telomerase; RI-4 analysis with TOP2A/MCM2, p16INK4a, cyclin E, and Ki-67; RI-3 analysis with TOP2A/MCM2, p16INK4a, and cyclin E, and RI-2 analysis with TOP2A/MCM2 and p16INK4a OR adjusted by age and HPV infection by oncogenic risk (HPV negative, HR, PHR, LHR, and UHR) ^a^reference category

## Discussion

Cervical cancer is a global health problem. Previously, our group reported the prevalence and distribution of HR-HPV infection in CC and precursor lesions in southern Mexico [18]. In this study, unlike the previous report, we were able to detect infections with multiple genotypes of both high- and low-risk HPV and found that the most frequent HR-HPV genotypes in CC were 16, 18, 45, 52, and 69. We found that 60% of CC samples were infected with a single, high-risk genotype, while the remaining 40% were infected with two or more genotypes. The frequency of multiple HPV infections has been documented in previous studies [24–28]. In this study, we used the INNO-LiPA method, which can detect 28 different HPV genotypes, allowing us to determine the distribution of the genotypes according to the severity of the cervical lesion.

Notably, we found that multiple HR-HPV infections are more frequent in LSIL (7.2%) and HSIL (14.9%) than in CC (7%), as are mixed infections (HR and PHR)—7.4% in LSIL, 7% in HSIL, and 0.3% in CC. Conversely, the frequency of HPV16 infection increased with lesion severity: 13.2% in LSIL, 13% in HSIL, and 42.3% in CC. These results suggest that HPV16, along with other HR-HPV genotypes, can initiate infection in early lesions and persist in lesions that progress to cancer until it is the only genotype detected (in approximately 40% of cases). Although it is not known whether co-infection with several high-risk genotypes enhances its carcinogenic effect, the high percentage of co-infections with HR-HPV is intriguing.

On the other hand, it is important to note that the application of an HPV preventive vaccine in Mexico began with the quadrivalent vaccine in 2008 for girls aged 11–13 [29]. In southern Mexico, particularly in the state of Guerrero, vaccination began with girls aged 11 to 13 in highly marginalized populations, and later extended to girls in schools and health centers. The women included in this study were 26 to 66 years old in 2013–2016, and thus it is inferred that they were not vaccinated, and therefore vaccination did not influence the observed frequencies of HPV 16, HPV 18, HPV 6, and HPV 11.

It is currently known that progression is a relatively rare event [30]. Many reports measured the expression of cellular biomarkers in various types of cervical samples to improve the efficiency of early diagnostic programs of CC, as well as the identification of premalignant lesions with a risk of progressing to CC, however, currently there is no biomarker capable of identifying lesions that will evolve to cancer. The analysis of viral and cellular biomarkers in a single non-invasive sample will help compare their efficiency and synergies to identify those that can be useful in this pursuit. In this study, we analyzed and characterized a panel of cellular biomarkers (TOP2A/MCM2, p16^INK4a^, cyclin-E, Ki-67, telomerase, ROS, and 8-OHdG) in single liquid-based cytology samples of LSIL, HSIL, and CC to determine the best candidates for identifying the cervical lesions that are more likely to progress to the next stage.

We found that TOP2A/MCM2, p16^INK4a^, cyclin-E, Ki-67, and telomerase increased according to lesion severity, and these observations coincide with other studies that reported biomarkers associated with the development of premalignant lesions and proposed its usefulness to identify the lesions that are most likely to progress to high-grade cervical disease and CC [31]. It has been reported that expression levels of p16^INK4a^ are useful for distinguishing HSIL from LSIL; however, they are probably not useful for distinguishing CIN 1 from non-CIN [7, 32]. Expression levels of Ki-67 and p16 have been suggested as useful for distinguishing cervical intraepithelial neoplasia (CIN) 3 and CIN 2, although Ki-67 showed less specificity than p16^INK4a^ [33–35]. In addition, it has been reported that telomerase expression was increased in LSIL and HSIL compared to NSIL samples [36], and increased expression of MCM2 and TOP2A (ProExC) was correlated with dysplasia and severity of cervical lesions [10, 11, 14]. On the other hand, we found that ROS the levels of 8-OHdG were higher in LSIL than in NSIL cases; however, their levels did not increase parallel to the progression of cervical lesions. This observation suggests that increased levels of ROS and 8-OHdG could be related to cervical pathogenesis because of HPV infection, but these molecules may not have an important biological role in the progression of cervical lesions. These observations agree with other studies that have reported that oxidative stress is associated with cervical carcinogenesis [17, 37, 38]; in one study, 8-OHdG levels were observed to stay constant among different SIL grades [37]. However, other studies reported that oxidative stress, and particularly 8-OHdG levels, increased in parallel to the severity of cervical lesion [17, 38].

We analyzed the expression of five cellular markers and their relation to SIL and cervical cancer development, and found that TOP2A/MCM2 staining is the best biomarker for discriminating between cervical lesion types, followed by p16^INK4a^, cyclin-E, Ki-67, and telomerase. However, the association increased only by grouping TOP2A/MCM2, p16^INK4a^, and cyclin-E (Tables 5 and 6). For the above, we proposed a panel of three cellular biomarkers (TOP2A/MCM2, p16^INK4a^, and cyclin-E), which, according to the statistical analysis and their function, are the most useful for evaluating the exacerbated proliferative activity of cervical cells, which is one of the earliest hallmarks of carcinogenesis. Other studies also indicated the usefulness of a biomarkers panel, based on the dual detection of p16^INK4a^/Ki-67 for the screening of cervical lesions induced by HPV [13, 39, 40].

Although many studies have analyzed the expression of these biomarkers, their efficiencies were not compared in a single liquid-based cytology sample, which is a less invasive method than a biopsy. In this paper, we propose a panel of cellular biomarkers that allow the identification, with high sensitivity and precision, of cervical lesions with a higher risk of progression to CC. This panel can be used rapidly, efficiently, and inexpensively to detect the presence of cervical lesions with a higher risk for progression to CC, in a single non-invasive sample from the squamocolumnar transformation zone, using liquid-based cytology. This method also has the advantage that the same cytological material can be used for HPV genotyping. Therefore, this paper provides strong evidence for the usefulness of these three biomarkers and the feasibility of their implementation in CC screening systems.

## Conclusions

The evaluation of TOP2/MCM2, p16^INK4a^, and cyclin E1 expression in a single liquid-based cytology sample is useful as a panel of biomarkers that allow the identification of cervical lesions with a higher risk for progression to CC. This method can be performed with high sensitivity and precision, and its implementation is thus feasible in CC screening systems.

## Supplementary Information

**Additional file 1: Table S1.** Expression levels of 8-OHdG and ROS according to histopathological diagnosis.

**Additional file 2: Table S1.** Principal component analysis (PCA) considering five cellular biomarkers.

**Additional file 3: Table S3.** Statistical power of the estimators obtained.

## Data Availability

The datasets used and/or analyzed during the current study are available from the corresponding author on reasonable request.

## Abbreviations

NSIL: Non-squamous intraepithelial lesions
LSIL: Low-grade squamous intraepithelial lesions
HSIL: High-grade squamous intraepithelial lesions
CC: Cervical cancer
PCNA: Proliferating cell nuclear antigen
MCM2: Minichromosome maintenance protein-2
TOP2A: DNA Topoisomerase II α
ROS: Reactive oxygen species
8-OHdG: 8-hydroxydeoxyguanosine
