# Supplementary material for: TOP2A/MCM2, p16INK4a, and cyclin E1 expression in liquid-based cytology: a biomarkers panel for progression risk of cervical premalignant lesions
Source: BMC Cancer. 2021 Jan 7;21:39. doi: 10.1186/s12885-020-07740-1 (PMC7792307; doi:10.1186/s12885-020-07740-1)
Supplement: Supplementary file 1 — Additional file 1: Table S1. Expression levels of 8-OHdG and ROS according to histopathological diagnosis. [file 12885_2020_7740_MOESM1_ESM.docx]

Table S1. Level expression of 8-OHdG and ROS according cyto/histopathological diagnosis.

|  | **NSIL**  Median  (IqR) | **LSIL**  Median  (IqR) | **HSIL**  Median  (IqR) | **CC**  Median  (IqR) |
| --- | --- | --- | --- | --- |
| 8-OHdG | 230248  (193225-283881) | 338123.3  (320780 – 357396.3) | 334338.5  (263545 – 385252) | 323504.9  (261561 – 349831) |
|  |  |  |  |  |
| ROS | 946  (408 – 1939) | 2333  (691 – 5953) | 1572  (708 – 2348) | 411.5  (249.25 – 820) |
|  |  |  |  |  |

IqR: interquartile range.
