# Supplementary material for: TOP2A/MCM2, p16INK4a, and cyclin E1 expression in liquid-based cytology: a biomarkers panel for progression risk of cervical premalignant lesions
Source: BMC Cancer. 2021 Jan 7;21:39. doi: 10.1186/s12885-020-07740-1 (PMC7792307; doi:10.1186/s12885-020-07740-1)
Supplement: Supplementary file 2 — Additional file 2: Table S1. Principal component analysis (PCA) considering five cellular biomarkers. [file 12885_2020_7740_MOESM2_ESM.docx]

Table S2. Principal component analysis (PCA) considering five cellular biomarkers.

| **Factors** | **Factor explanation** |
| --- | --- |
| TOPII alpha/MCM2 | 0.938 |
| Ciclina E1 | 0.913 |
| p16INK4a | 0.914 |
| Ki-67 | 0.893 |
| Telomerasa | 0.889 |
