# Supplementary material for: TOP2A/MCM2, p16INK4a, and cyclin E1 expression in liquid-based cytology: a biomarkers panel for progression risk of cervical premalignant lesions
Source: BMC Cancer. 2021 Jan 7;21:39. doi: 10.1186/s12885-020-07740-1 (PMC7792307; doi:10.1186/s12885-020-07740-1)
Supplement: Supplementary file 3 — Additional file 3: Table S3. Statistical power of the estimators obtained. [file 12885_2020_7740_MOESM3_ESM.docx]

**Table S3. Statistical power of the estimators obtained.**

|  | **NSIL** | | **LSIL** | | | | **HSIL** | | | | **CC** | | | |
| --- | --- | --- | --- | --- | --- | --- | --- | --- | --- | --- | --- | --- | --- | --- |
|  | **N** | **P** | **N** | **N2/N1** | **OR** | **1-β*** | **N** | **N2/N1** | **OR** | **1-β*** | **N** | **N2/N1** | **OR** | **1-β*** |
| **TOP2A/MCM2** | 79 | 0.4684 | 208 | 2.6329114 | 22.5 | 1.0 | 35 | 0.44303797 | 900.8 | 1.0 | 42 | 0.53164557 | 2484 | 1.0 |
| **p16INK4a** | 79 | 0.5822 | 208 | 2.6329114 | 10.1 | 1.0 | 35 | 0.44303797 | 138.1 | 1.0 | 42 | 0.53164557 | 320.6 | 1.0 |
| **Cyclin E** | 79 | 0.5444 | 208 | 2.6329114 | 10.0 | 1.0 | 35 | 0.44303797 | 109.9 | 1.0 | 42 | 0.53164557 | 533.0 | 1.0 |
| **Ki-67** | 79 | 0.5822 | 208 | 2.6329114 | 6.7 | 1.0 | 35 | 0.44303797 | 121.4 | 1.0 | 42 | 0.53164557 | 522.8 | 1.0 |
| **Telomerase** | 79 | 0.6456 | 208 | 2.6329114 | 13.7 | 1.0 | 35 | 0.44303797 | 85.7 | 0.997 | 42 | 0.53164557 | 165.9 | 0.999 |
| **RI-5** | 79 | 0.1898 | 208 | 2.6329114 | 58 | 1.0 | 35 | 0.44303797 | 4012 | 1.0 | 42 | 0.53164557 | 8290 | 1.0 |
| **RI-4** | 79 | 0.21512 | 208 | 2.6329114 | 29.5 | 1.0 | 35 | 0.44303797 | 1482 | 1.0 | 42 | 0.53164557 | 3878 | 1.0 |
| **RI-3** | 79 | 0.2405 | 208 | 2.6329114 | 40.2 | 1.0 | 35 | 0.44303797 | 2924 | 1.0 | 42 | 0.53164557 | 5913 | 1.0 |
| **RI-2** | 79 | 0.4810 | 208 | 2.6329114 | 24.5 | 1.0 | 35 | 0.44303797 | 1290 | 1.0 | 42 | 0.53164557 | 3020 | 1.0 |
| **ROS**** | 129 | 0.7054 | 212 | 1.6434109 | 1.8 | 0.6122 | 29 | 0.2248062 | 1.2 | 0.068 | 32 | 0.24806202 | 0.5 | 0.397 |
| **8-OHdG** | 93 | 0.3226 | 170 | 1.827957 | 4.4 | 0.999 | 26 | 0.27956989 | 3.6 | 0.80 | 32 | 0.34408602 | 5.3 | 0.971 |

NSIL, non-squamous intraepithelial lesions (control); LSIL, low-grade squamous intraepithelial lesions; HSIL, high-grade squamous intraepithelial lesions; CC, cervical cancer; OR, odds ratio; RI, risk index

RI-5 analysis with TOP2A/MCM2, p16INK4a, cyclin E, Ki-67, and telomerase; RI-4 analysis with TOP2A/MCM2, p16INK4a, cyclin E, and Ki-67; RI-3 analysis with TOP2A/MCM2, p16INK4a, and cyclin E; RI-2 analysis with TOP2A/MCM2 and p16INK4a.

*1-β: Statistical power. For the calculation of statistical power, the comparison of proportions of two independent samples based on the likelihood ratio in terms of odds ratios was used. With two-tailed test and α = 0.05

**To increase the reliability of the ROS estimators, for a statistical power of 0.80, in LSIL it is required to increase the total sample size to 506 with 253 individuals per group. While for CC a total sample of 284 individuals is needed, 142 for each group.
